# Physical fitness assessment in wheelchair basketball: A mini-review

**DOI:** 10.3389/fspor.2022.1035570

**Published:** 2022-12-09

**Authors:** Luca Petrigna, Simona Pajaujiene, Giuseppe Musumeci

**Affiliations:** ^1^ Department of Biomedical and Biotechnological Sciences, Section of Anatomy, Histology and Movement Science, School of Medicine, University of Catania, Catania, Italy; ^2^ Department of Coaching Science, Lithuanian Sports University, Kaunas, Lithuania; ^3^ Research Center on Motor Activities (CRAM), University of Catania, Catania, Italy; ^4^ Department of Biology, Sbarro Institute for Cancer Research and Molecular Medicine, College of Science and Technology, Temple University, Philadelphia, PA, United States

**Keywords:** standard operating procedure (SOP), evaluation, disability, sport, performance

## Abstract

**Introduction:**

Wheelchair basketball (WB) is a Paralympic sport ideated for people with motor disabilities, and the research on this topic still requires attention. It is fundamental to evaluate physical fitness characteristics with appropriate tests and standardized routines to plan and monitor the training. Considering that a standard operating procedure is a document that makes the test battery replicable, the objective of the present study was to review the literature on physical fitness assessment in WB players and to create a standard operating procedure.

**Methods:**

Studies were collected from different databases, and after a screening process, data were discussed narratively.

**Results:**

Only 18 articles met the eligibility criteria. The test batteries presented similarities in different studies.

**Conclusion:**

The suggested standard operating procedure consists of 10-min warm-up followed by handgrip evaluation (only if the instrument is available), 20-m sprint test, maximal pass, modified push-up, back scratch test, and the Yo-Yo intermittent recovery test adapted test Version 1.

## Introduction

The Paralympic Games, an event parallel to the Olympic Games, is the most important sports competition for people with physical disabilities (amputation or limb deficiencies), nervous impairments (cerebral palsy or spinal cord-related disability), visual and intellectual impairment, or other disabilities (not included in the previous classifications) (1). One sport of the Paralympic program is wheelchair basketball (WB), and it includes athletes with motor disabilities (2). WB is characterized by high-intensity activities alternated with long recovery periods; it generally requires high levels of aerobic and anaerobic fitness (3). In the high-intensity period, WB athletes repeat short, intense exercise bouts that include rapid acceleration and deceleration, dynamic position changes, and maintaining or obtaining one’s position on the court (4).

To plan and monitor the training and know the level of the athletes, it is fundamental that a periodic evaluation is performed. Field tests are easier, faster, and cheaper to administer than laboratory evaluation (5), making them ideal during the season. As was done in other Paralympic sports (6, 7), the evaluation routine could be proposed as a standard operating procedure (SOP). An SOP is a document that provides details of a process to allow for a correct repetition of the various steps, to make the testing session replicable, and to ensure the normalization of data (8). For the above reasons, the objective of this study was to review the literature to propose an SOP to evaluate physical fitness with field tests in WB players.

## Materials and methods

Participants included were elite athletes practicing WB. No limitations were adopted related to the intervention. For comparison, physical fitness had to be evaluated with field tests. Only English-written original manuscripts were included.

Studies were collected from PubMed, Web of Science (WoS), and Scopus databases, if published before July 22, 2022. The keywords wheelchair basketball, wheel chair basketball, wheelchair basket and physical fitness, sports physiology, and performance analysis were matched by adopting the Boolean operator AND/OR.

Field tests adopted to evaluate health-related physical fitness components (cardiorespiratory endurance, muscular strength and endurance, flexibility, and body composition) as classified by Caspersen et al. (9) were considered.

## Results and discussion

A total of 349 (155 PubMed, 166 WoS, 28 Scopus) articles were found. After duplicate removal, 292 manuscripts were detected. A total of 18 studies were analyzed after the eligibility criterion selection. The sample of the studies included ranged from 8 to 61 athletes, and the adopted tests were heterogeneous. More details about the analyzed physical fitness test sets are provided in Table 1.

**Table 1:** Information of the included studies.

Reference	Number [f] (m)	Endurance	Muscle strength and endurance	Flexibility/body composition
Bergamini et al. (10)	12 (2) [10]	20 m sprint		
Cavedon et al. (11)	52 [7] (45)	5/20 m B, suicide	MP	
Cavedon et al. (12)	39 (13) 26	5/20 m B; suicide	MP	
Gil et al. (13)	13 [13]	20 m sprint B-NB; Yo-Yo	Handgrip, MP, MBT	
Granados et al. (14)	19	5/20 m sprint B-NB; Yo-Yo	MP, MBT, handgrip	ST
Hutzler et al. (15)	11 (11)	6 min; 428 m racing trials; slalom parcourse		
Iturricastillo et al. (16)	12	Yo-Yo		
Iturricastillo et al. (17)	8	5/20 m sprints B-NB; Yo-Yo	MP, MBT, handgrip	ST
Iturricastillo et al. (18)	9	Repeated sprint ability; 20 m sprint		
Iturricastillo et al. (19)	13 [13]	20 m sprint		
Marszałek et al. (20)	61 [61]	3/5/10/20 m sprint; 30 s sprint; 10 × 5 m sprint	MP, MBT (3 kg), handgrip	
Tachibana et al. (21)	26 [26]	20 m sprint; Yo-Yo	MP	
Yanci et al. (22)	16 (2) [14]	Sprint B-NB; Yo-Yo	Handgrip, MP	ST
Yüksel and Sevindi (23)	21 (21)	20 m sprint; slalom B-NB; 6 min	Modified sit-up, modified abdominal endurance, modified push-up, handgrip	Back scratch test
Vanlandewijck et al. (24)	46 [46]	20 m shuttle run test, 30 s sprint		
Wang et al. (25)	37 (21) [16]		Dynamometer	ROM
Weissland et al. (26)	16 (2) [14]	MFT, modified version		ST
Weissland et al. (27)	18 [2] (16)	MFT, 30–15 intermittent fitness test		

B, with the ball; MFT, incremental multistage field test; MP, maximal pass; MBT, medicine ball throw; ST, skinfold thicknesses; NB, without the ball; ROM, range of motion.

### Cardiorespiratory endurance

The most adopted test (*n* = 6) to evaluate cardiorespiratory endurance was the Yo-Yo intermittent recovery adapted test Version 1. The protocol adopted was previously published (28), and it was adapted to the athlete's conditions (22). The test consisted of 10-m wheelchair shuttle runs with increased velocities, alternated with 10 s of active recovery until exhaustion (the athlete failed twice to reach the front line in time or felt unable to cover another shuttle at the dictated speed). The total distance covered during the test was measured. This test is included in our SOP not only because it is the most used test but also because the Yo-Yo test is, generally, a valid and simple method to collect data on the capacity of the athlete to perform repeated intense exercise (29). Other adopted tests were the suicide test, the incremental multistage field test and its modified version (*n* = 2), the 20-m shuttle run test, the repeated sprint ability test (12 × 20 m every 20 s or 10 × 5 m version), and the 30–15 intermittent fitness test (*n* = 1). Other tests adopted to evaluate cardiorespiratory endurance were 6 min continuous wheeling around an elliptical concrete track of 107 m circumference (*n* = 2), 428-m racing trial (*n* = 1), and a unique wheelchair slalom parcourse, including 15 direction changes and four short sprints of 10 m each (*n* = 1).

Widely adopted was the 20-m sprint test (*n* = 11). The protocol of Gorostiaga et al. (30) was followed by four authors: it consists of three maximal wheelchair sprints alternated with 120 s of rest, with the best value adopted for analysis. The participants were placed at 0.5 m from the starting point and began when they felt ready. An option was to detect split times at 5 (31) and 20 m (24). Two maximal sprints were adopted in two studies (17, 19, 21). Cavedon et al. (11) asked to start the test to a proper signal. The same protocol was proposed in four studies (11, 13, 14, 22) with the ball, following the official game rules for dribbling (31). A similar protocol was adopted by Cavedon et al. (12). Other distances adopted were 5 (*n* = 5), 3, and 10 m (*n* = 1). Less adopted sprint evaluation (*n* = 2) consisted of a 30-s sprint test.

### Muscle strength and endurance

The handgrip evaluation was used in five studies. It was measured in the dominant hand, with the arm in extension and in the vertical axis. The participants performed the test seated in their wheelchairs with the arm fully extended and not touching the wheelchairs (32). The testing protocol consisted of three maximal isometric contractions for 5 s, with a rest period of at least 60 s. The highest value was used for analysis. The dynamometer had to be squeezed as hard as possible. Visual feedback on the recorded strength was provided. Bilateral handgrip was measured by Marszalek et al. (20), and the combination of the value for the right and left hands was recorded. This test presents validity and reliability (33, 34), making it ideal for strength evaluation. Muscle strength was also measured at the shoulder, elbow, and wrist joints of the dominant arm using a dynamometer (25).

The maximal pass was adopted in five studies. Front wheels behind a line, in a stationary position (one researcher holds the basketball wheelchair still), participants had to pass a basketball ball with a two-arm overhand throw as far as possible. The distance between the participant and where the ball hit the floor was measured (meters). The end score was the average distance of five passes. The tested domain was passing (explosiveness) (31). A similar version was also used (11, 12) with a basketball chest pass using both arms as symmetrically as possible (20). Tachibana et al. (21) adopted a similar procedure but evaluated three types of passes: chest, baseball, and hook pass. The medicine ball throw was also adopted in five studies (four studies adopted a 5 kg ball, while one study adopted a 3 kg ball), but it was not suggested in the SOP because it requires specific equipment.

Muscle endurance was evaluated with the modified sit-up, modified abdominal endurance, and modified push-up (23). Among these tests, the modified push-up is the one that can be adopted in the SOP because it evaluates the strength of the upper body and is an indicator of different health outcomes, such as perceived health, mobility, and disability (35). Furthermore, this test presents reliability and a test–retest variation that is small (36).

### Flexibility

To evaluate shoulder flexibility, the back scratch test (*n* = 1) has been adopted following a previously published protocol (37). From a sitting position with the back vertical, the distance (centimeters) was measured between the second finger of the two hands placed behind the back: if the fingers were in contact, the value was 0. One arm reached over the shoulder and the other one down the back. This test is included in the Brockport Physical Fitness test manual (38), making it ideal for people with disabilities. The range of motion of the shoulder, elbow, and wrist joints was also measured using a double-arm goniometer (*n* = 1), but since it requires specific tasks to be adopted, it is not suggested in our SOP.

### Anthropometric variables

Skinfold thicknesses (in millimeters) were measured at four sites (triceps, subscapular, abdominal, and suprailiac) in five studies. Skinfold thickness is generally a cheap, valid, and accurate body composition measurement (39). Rather than using the four points, in the SOP, a two-point skinfold evaluation could be adopted because it is simpler and faster to administer (40).

### Standard operating procedure

The SOP that could be adopted to evaluate WB athletes starts with the anthropometric and two-point skinfold evaluation. After that, participants do a 10-min warm-up, as adopted in different studies (20, 21), including sprints, ball-handling, passing, shooting, propelling the basketball wheelchair around the court, and the dynamic stretching of upper limbs and trunk. The intensity should be low to medium, and stretching exercises should be included (11, 12). The following tests could be proposed: handgrip evaluation (only if the instrument is available); 20-m sprint test, maximal pass, modified push-up, back scratch test, and the Yo-Yo intermittent recovery adapted test Version 1. Participants have to be instructed to perform all tests at maximum intensity with adequate rest between tests (15 min of rest time before the Yo-Yo test) and the possibility to practice before the test trial (11, 12). Considering the study of Cavedon et al. (11), the sequence of tests could be handgrip evaluation, 20-m sprint, maximal pass, Yo-Yo test, with at least 2-min rest between the tests. During data collection, each participant used their basketball wheelchair. No indication for the propulsion strategy is given, and players freely use their push rate and modality (27).

An important limitation of the present study is the poor number of included studies, but this highlights the importance of future original articles on this topic. The proposed SOP is in its first step in which published articles are analyzed, and the conclusions are made from other studies’ results. In the next step, the validity and feasibility of the SOP should be analyzed with original studies.

## Conclusion

The creation of an SOP for the physical fitness evaluation of WB athletes will help researchers further develop this topic, working in the same direction all over the world. In this way, it will be possible to compare the findings and create normative data according to the players’ level and experience. The SOP will also be useful for coaches to compare their team data to better understand the level and plan the training appropriately.
